# Type 2 diabetes morbidity, mortality and its associated risk factors across Brazilian regions: findings from the Global Burden of Disease Study 2021

**DOI:** 10.20945/2359-4292-2026-0052

**Published:** 2026-05-17

**Authors:** Paula Portal Teixeira, Tatiana Duque-Cartagena, Lucas Scotta Cabral, Bárbara Niegia Garcia de Goulart, Rodrigo dos Reis, Fernando Gerchman, Verônica Colpani, Deborah Carvalho Malta, Yvonne Yiru Xu, Maria Inês Schmidt, Rita Mattiello, Bruce Bartholow Duncan

**Affiliations:** 1 Programa de Pós-graduação em Ciências Médicas: Endocrinologia, Universidade Federal do Rio Grande do Sul, Porto Alegre, RS, Brasil; 2 Programa de Pós-graduação em Epidemiologia, Faculdade de Medicina, Universidade Federal do Rio Grande do Sul, Porto Alegre, RS, Brasil; 3 Programa de Pós-graduação em Cardiologia e Ciências Cardiovasculares, Universidade Federal do Rio Grande do Sul, Porto Alegre, RS, Brasil; 4 Centro de Avaliação de Tecnologias em Saúde, Hospital Sírio-Libanês, São Paulo, SP, Brasil; 5 Programa de Pós-graduação em Saúde Pública, Universidade Federal de Minas Gerais, Belo Horizonte, MG, Brasil; 6 Institute for Health Metrics and Evaluation, University of Washington, Seattle, USA

**Keywords:** Diabetes mellitus, global burden of disease study, risk factors, Brazil

## Abstract

**Objective:**

To describe Brazilian national and regional trends in type 2 diabetes mellitus (T2DM) prevalence, incidence, burden, and exposure to T2DM risk factors.

**Materials and methods:**

We sourced the Global Burden of Diseases Study (GBD) 2021 to obtain estimates and trends of T2DM deaths, incidence, prevalence, Years of Life Lost (YLLs), Years Lived with Disability (YLDs), and Disability Adjusted Life Year (DALYs) in Brazil and its regions. We present crude and age-standardized metrics, as well as the exposure to T2DM risk factors between 1990 and 2021.

**Results:**

The national age-standardized prevalence of T2DM increased by 37.4% (95% UI 32.7 to 42.6) and the incidence by 32.3% (95% UI 27.6 to 37.7) from 1990 to 2021. Age-standardized deaths by T2DM decreased by 18.0% (95% UI -21.4 to -15.3), and the accompanying YLLs by 22.8% (95% UI -25.8 to -20.2). YLDs increased by 35.4% (95% UI 29.1 to 41.3), while DALYs’ rates reduced by 3.1% (95% UI -8.2 to 1.7) since 1990. The Northeast region showed higher age-standardized prevalence, incidence, YLLs, and YLDs in 2021, while the North region had the most pronounced increases. T2DM prevalence increased consistently, alongside rises in exposure to sugar-sweetened beverage consumption and high BMI. While smoking exposure declined in all regions, low physical activity and diets high in red and processed meat increased over time.

**Conclusion:**

T2DM burden in Brazil is growing due to the increasing exposure to T2DM risk factors. Greater emphasis on prevention and public policies focusing on reducing risk factors and inequalities can reduce T2DM burden in Brazil.

## INTRODUCTION

Type 2 diabetes mellitus (T2DM) is a non-communicable disease that affects mainly adults and is characterized by insulin resistance and reduced pancreatic insulin secretion ^(1)^. The condition is a major cause of blindness, kidney failure, heart attacks, stroke, and lower limb amputation, especially in those with poor glycemic control ^(2)^. According to the NCD Risk Factor Collaboration (NCD-RisC), in 2022, age-standardized diabetes prevalence in the world was 13.9% for women and 14.3% for men, representing around 828 million adults living with diabetes worldwide ^(3)^. Projections from the Global Burden of Diseases, Injuries and Risk Factors Study (GBD) show that, despite significant improvements envisioned in its treatment, T2DM can become Brazil´s leading cause of disease burden, represented by disability-adjusted life year (DALYs), in 2050 ^(4)^.

Previous studies evaluating the burden of T2DM in Brazil have focused exclusively on prevalence ^(5-7)^, incidence ^(8)^, mortality ^(9-11)^, risk factors ^(12)^, or hospitalizations ^(10)^, while some, based on GBD data, focused on DALYs ^(13)^ or economic outcomes ^(14)^. However, comprehensive assessments of the aggregate burden of non-fatal and fatal outcomes and risk factors of T2DM across the country are scarce. Additionally, some studies that relied only on self-reported T2DM data ^(5,6,11,12)^ underestimated the total prevalence of the condition and considered only those aged 18 and over ^(8,11)^, overlooking the increasing incidence of T2DM in younger populations. Finally, none of these studies have gone beyond 2019, making it pertinent to incorporate the available and more current data.

To help inform Brazilian stakeholders in confronting the country’s diabetes epidemic, we aimed to provide a comprehensive picture of the T2DM burden in Brazil and its macro-regions. We describe national and regional trends in prevalence, incidence, and burden, and trends in exposure to underlying potentially modifiable behavioral, environmental, and metabolic diabetes risk factors from 1990 to 2021.

## MATERIALS AND METHODS

### Overview and definitions

This study adheres to the Guidelines for Accurate and Transparent Health Estimates Reporting (GATHER) statement, ensuring quality reporting and transparency in assessing health estimates ^(15)^. The GBD is a multinational collaboration that synthesizes primary and secondary epidemiological data to comprehensively evaluate the health impact of diseases, injuries, and risk factors within diverse populations and geographical contexts worldwide. All steps related to the GBD 2021 estimation process, from compiling data sources through data identification and extraction, data adjustment, case definitions, and metric estimations, have been previously described in greater detail ^(16-18)^. This manuscript was produced as part of the GBD Brazil Network ^(19)^ following the GBD Protocol.

For this analysis, we downloaded data directly from the GBD Results Tool (http://ghdx.healthdata.org/gbd-results-tool). We focused on estimates of T2DM deaths, prevalence, incidence, Years of Life Lost due to premature mortality (YLLs), Years Lived with Disability (YLDs), and Disability-adjusted Life Years (DALYs) in Brazil and its regions (North, Northeast, Central-West, Southeast, and South), presenting the overall percent change of each estimate from 1990 to 2021. We also described the percentage change in age-standardized death rates disaggregated by sex to assess whether the estimates apply uniformly across male and females. All estimates are shown in rate per 100,000 habitants as age-standardized and in crude form (encompassing all age groups) and accompanied by 95% uncertainty intervals (UIs). We also present estimates on the percent change in population exposure to several major T2DM risk factors for Brazil overall and by region between 1990 and 2021. All graphs presented in this manuscript were generated using The R Foundation for Statistical Computing Platform, version 4.1.2 (2021).

#### Diabetes definition

The GBD study categorizes causes of death and disability into four hierarchical levels, from a broader to a more detailed level, and diabetes mellitus is considered a level 3 cause. Its burden is the combination of type 1 (T1DM) and type 2 (T2DM) diabetes mellitus. The reference case definition for overall diabetes is the presence of a fasting plasma glucose (FPG) ≥126 mg/dL (7 mmol/L) or the report of drug treatment for diabetes (insulin or anti-diabetic drugs) ^(20)^. In order to standardize data with different definitions of diabetes, when data on an oral glucose tolerance test (OGTT) and glycated hemoglobin are also reported, these values are transformed into FPG equivalents.

### Diabetes data sources and estimation

To estimate T2DM mortality in Brazil, GBD 2021 gathered data on deaths from 40 sources, including vital registration in ICD-10 codes, which are all provided through the Global Health Data Exchange (GHDx) (https://ghdx.healthdata.org/). To do so, GBD conducts systematic literature reviews of published studies, which it aggregates to data from government and international organization websites, primary data sources, and contributions by country GBD collaborators and the World Health Organization (WHO). Deaths from diabetes, identified by the International Classification of Diseases (ICD) version 10 (E10–E14 and P70.2), were modelled to generate diabetes mortality estimates using a Bayesian geospatial regression analysis called Cause of Death Ensemble model (CODEm) ^(16)^. Estimates were obtained by age, sex, year, and geography. Additionally, whenever deaths obtained from GBD sources were coded to an implausible underlying cause of death or unspecified diabetes types, corrections and redistributions by manual and statistical methods were performed to redistribute these deaths to probable underlying causes, including T2DM ^(16)^.

Type-specific diabetes deaths were estimated using vital registration data. It was assumed that all deaths under age 15 were due to T1DM. Once the data had been gathered and cleaned, estimates were modeled over the years and across regions ^(16)^. To estimate premature mortality, the GBD employed the metric Years of Life Lost (YLLs), multiplying the number of deaths from T2DM in each age group by the reference life expectancy at the average age of death for those who die in that age group.

For non-fatal estimation, GBD obtained data from 17 sources in Brazil, including systematic reviews that only consider population representative surveys obtained directly or through publications. After data compiling and initial adjustments, prevalence data were summarized across Brazil’s regions using a hierarchical Bayesian meta-regression modeling tool (DisMod-MR 2.1), which integrated the various metrics to provide consistent prevalence, incidence, and mortality estimates ^(18)^. The model considers covariates and the location hierarchy to inform locations, years, ages, and sex where data is missing. For non-fatal outcomes, GBD derived estimates of T2DM by deducting the estimated prevalence of T1DM from the overall estimated diabetes mellitus prevalence. Estimates of prevalence and incidence were split between T1DM and T2DM, with all cases <15 years of age assumed to be T1DM, and are not the focus of this manuscript ^(20)^.

The GBD estimated the burden due to disability (morbidity) through the metric Years Lived with Disability (YLDs), obtained by the product of the prevalence of each sequelae of T2DM (neuropathy, diabetic foot, lower limb amputation, vision loss due to retinopathy, as well as living with diabetes) and its corresponding disability ^(18,21)^. The disability weights, on a scale from 0 to 1, represent the magnitude of health loss in the presence of these complications and were calculated separately for each sequelae. The sum of YLDs across sequelae and age/sex strata produced the total YLDs for T2DM for a given year and location. Finally, Disability-Adjusted Life Years (DALYs) were obtained through the sum of YLLs and YLDs and represent total health loss due to premature mortality or morbidity from T2DM ^(18)^.

#### Risk factors estimation

The GBD identified risk factors associated with T2DM through systematic reviews and meta-analyses using meta-regression procedures. Based on the exposure levels to each risk factor, a Bayesian meta-regression model (DisMod-MR 2.1) and spatiotemporal Gaussian process regression model (ST-GPR) were used to estimate the exposure levels for the entire population over time. The relative risk for each risk factor was then used to calculate the excess disability and death that occurred above the theoretical minimum exposure level (TMREL), representing the level of risk exposure that minimizes the risk of T2DM at the population level. The level of exposure to risk factors was estimated by their Summary Exposure Value (SEV). For continuously measured risk factors, the SEV weighs the prevalence of different levels of the risk factor by the severity of the burden caused at each level. GBD 2021 reported SEVs on a scale from 0 to 100%. Though the comparison of the absolute values of SEVs across risk factors is of limited value, tendencies in SEVs across time and space facilitate comparisons of risk factor exposures ^(17)^.

## RESULTS

In 2021, Brazil presented an age-standardized diabetes prevalence of 5095.8 per 100,000 habitants (95% UI 4578.5 to 5678.2) and an age-standardized diabetes incidence of 279.1 (95% UI 251.6 to 309.3) per 100,000. From 1990 to 2021, these frequencies increased by 37.4% (95% UI 32.7 to 42.6) and 32.3% (95% UI 27.6 to 37.7), respectively. However, when considering all age groups, the changes from 1990 to 2021 were notably higher, 135.5% (95% UI 127.5 to 145.0) and 113% (95% UI 102.8 to 123.5), respectively (**Table 1**).

**Table 1.** Burden of T2DM in Brazil and its regions from 1990 to 2021

	All ages		Age-standardized	
	Rate per 100,000, 2021 (95% UI)	Change 1990–2021 (%)	Rate per 100,000, 2021 (95% UI)	Change 1990–2021 (%)
Prevalence				
Brazil	5895.9 (5290.8 to 6575.9)	135.5 (127.5 to 145)	5095.8 (4578.5 to 5678.2)	37.4 (32.7 to 42.6)
North	4783.0 (4312.9 to 5324.6)	176.1 (164.7 to 188.5)	5566.9 (5020.0 to 6173.2)	57.1 (50.9 to 63.4)
Northeast	5982.3 (5414.4 to 6628.3)	153.6 (144.7 to 163)	5816.3 (5261.1 to 6430.2)	55.5 (49.9 to 61)
Central-West	5869.7 (5277.5 to 6518.9)	180.5 (167.7 to 193.6)	5335.0 (4806.3 to 5888.8)	44 (38.4 to 50.0)
Southeast	5910.6 (5267.4 to 6666.4)	107.9 (98.3 to 119)	4638.6 (4132.9 to 5225.9)	20.3 (14.9 to 26.2)
South	6414.3 (5731.9 to 7192.9)	174.6 (161.6 to 189.1)	4916.2 (4403.0 to 5498.6)	50.4 (43.9 to 58)
**Incidence**					
Brazil	326.1 (293.3 to 362.6)	113.0 (102.8 to 123.5)	279.1 (251.6 to 309.3)	32.3 (27.6 to 37.7)
North	292.9 (263.9 to 324.4)	170.1 (156.7 to 185)	303.4 (273.3 to 334.0)	55.9 (49.3 to 63)
Northeast	336.2 (304.7 to 371.1)	147.3 (137 to 159.1)	317.9 (288.1 to 350.0)	51.4 (45.4 to 57.8)
Central-West	338.9 (304.1 to 376.8)	145.1 (130.6 to 162.9)	288.4 (260.0 to 319.9)	36.6 (29.9 to 43.9)
Southeast	316.6 (281.8 to 355.5)	79.6 (68.9 to 91.6)	252.5 (225.6 to 283.0)	14.3 (8.3 to 20.7)
South	347.4 (309.2 to 388.3)	134.5 (121.2 to 150.5)	271.4 (242.6 to 302.4)	41.3 (35.1 to 48.7)
**Deaths**					
Brazil	28.7 (26.2 to 30.2)	20.5 (14.2 to 25.8)	25.8 (23.5 to 27.3)	-18.0 (-21.4 to -15.3)
North	22.7 (20.6 to 24.6)	88.2 (71.6 to 106.3)	31.7 (28.5 to 34.4)	26.1 (16.2 to 36.3)
Northeast	32.0 (29.1 to 34.1)	51.7 (41.8 to 61.3)	32.2 (29.3 to 34.3)	-2.8 (-8.0 to 1.8)
Central-West	21.1 (19.1 to 22.6)	30.5 (20.8 to 39.5)	22.2 (19.9 to 23.8)	-7.0 (-13.1 to -0.9)
Southeast	27.7 (24.8 to 29.5)	-3.6 (-10.2 to 2.3)	22.1 (19.7 to 23.6)	-35.0 (-38.9 to -31.2)
South	32.8 (29.7 to 35.0)	36.9 (26.5 to 45.3)	25.9 (23.3 to 27.7)	1.9 (-4.8 to 8.3)
**Years of Life Lost (YLL)**					
Brazil	612.5 (574.0 to 636.7)	61.3 (51.9 to 70.3)	536.7 (501.5 to 558.5)	-22.8 (-25.8 to -20.2)
North	521.1 (479.9 to 561.8)	161.2 (133.9 to 192.2)	665.2 (610.1 to 717.7)	22.4 (12.5 to 32.5)
Northeast	676.3 (632.0 to 712.8)	146.0 (125.4 to 169.8)	670.9 (626.1 to 707.4)	-6.3 (-11.1 to -1.6)
Central-West	478.6 (444.1 to 509.7)	66.7 (53.4 to 81.3)	460.6 (423.5 to 491.0)	-12.8 (-18.5 to –7.0)
Southeast	592.0 (548.4 to 628.9)	17.6 (9.9 to 25.2)	463.8 (428.9 to 492.8)	-39.3 (-42.6 to -35.6)
South	673.0 (621.6 to 715.0)	55.5 (44.0 to 66.0)	515.9 (475.7 to 548.1)	-6.7 (-12.8 to -0.5)
**Years Lives with Disability (YLDs)**					
Brazil	564.5 (391.3 to 771.9)	95.2 (86.0 to 105.1)	486.9 (337.6 to 664.1)	35.4 (29.1 to 41.3)
North	449.9 (316.3 to 616.1)	129.1 (116.7 to 141.5)	533.4 (374.3 to 728.1)	53.4 (46.3 to 60.7)
Northeast	571.0 (393.8 to 778.6)	110.3 (99.4 to 120.5)	557.1 (384.7 to 757.1)	52.8 (45.4 to 59.2)
Central-West	556.3 (383.8 to 751.8)	128.0 (113.4 to 143.1)	508.0 (349.8 to 683.2)	41.3 (33.2 to 48.6)
Southeast	568.9 (392.7 – 780.1)	73.5 (63.1 to 84.2)	443.4 (306.3 to 607.7)	19.6 (12.5 to 26.6)
South	617.2 (426.0 to 841.9)	120.0 (106.4 to 134.1)	469.0 (325.3 to 638.4)	45.6 (37.1 to 54.1)
**Disability Adjusted Life Years (DALYs)**					
Brazil	1177.0 (999.4 to 1387.0)	79.2 (69.8 to 87.8)	1023.6 (870.9 to 1203.7)	-3.1 (-8.2 to 1.7)
North	971.0 (824.8 to 1146.3)	155.8 (139.8 to 175.4)	1198.7 (1022.4 to 1410.5)	34.8 (27.2 to 41.4)
Northeast	1247.4 (1068.0 to 1456.4)	143.0 (128.4 to 160.2)	1228.0 (1051.5 to 1431.9)	13.5 (6.9 to 19.6)
Central-West	1034.9 (867.4 to 1241.5)	97.6 (85.4 to 109.5)	968.6 (815.0 to 1155.7)	9.2 (3.0 to 14.8)
Southeast	1160.9 (980.3 – 1385.4)	40.5 (31.7 to 48.6)	907.2 (766.2 to 1082.3)	-20.3 (-25.6 to –15.0)
South	1290.2 (1096.4 to 1524.5)	81.1 (71.3 to 90.7)	984.9 (835.9 – 1161.5)	13.2 (7.3 to 19.1)

UI: Uncertainty intervals.

Regionally, the Northeast stood out with the highest age-standardized prevalence of T2DM in 2021, 5816.3 per 100,000 habitats (95% UI 5261.1 to 6430.2), as well as the highest incidence, at 317.9 (95% UI 288.1 to 350.0), 14% greater than that for Brazil overall. In contrast, the Southeast region had the lowest age-standardized prevalence, at 4638.6 (95% UI 4132.9 to 5225.9), and incidence, at 252.5 (95% UI 225.6 to 283.0). The North region showed the most pronounced increase in the age-standardized prevalence of T2DM from 1990 to 2021, 57.1% (95% UI 50.9 to 63.4), 53% greater than that for Brazil overall and contrasting with the Southeast region, which experienced the smallest increase, 20.3% (95% UI 14.9 to 26.2). Considering all age estimates, the South region showed the highest prevalence and incidence, and the North region was the lowest for both estimates **(Table 1)**.

**Supplementary Figure 1.**
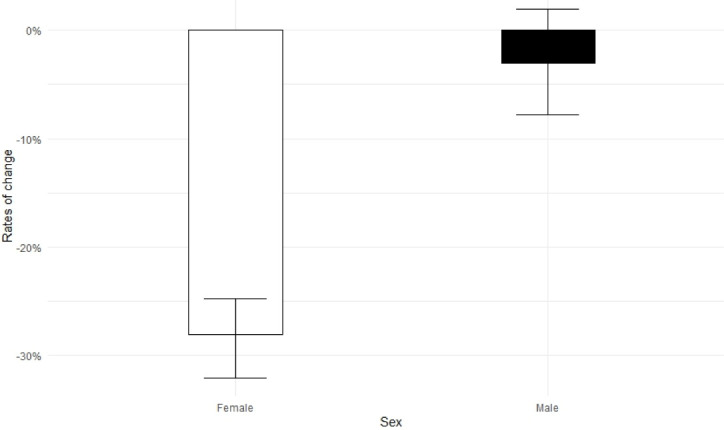
Rate of change in age-standardized mortality attributed to T2DM in Brazil, according to sex, from 1990 to 2021.

The age-standardized mortality rate in Brazil in 2021 was 25.8 (95% UI 23.5 to 27.3) per 100,000 habitants by 2021, reflecting an 18% decrease (95% UI -21.4 to -15.3) since 1990. YLLs were 536.7 (95% UI 501.5 to 558.5) per 100,000 inhabitants, a 22.8% decrease (95% UI -25.8 to -20.2) compared to 1990. When age-standardized mortality was disaggregated by sex, females experienced a 28% reduction in age-standardized deaths (-28.0%; 95% UI -32.1 to -24.7), whereas males exhibited only a 3% reduction (-3.0%; 95% UI -7.8 to 1.9) (**Supplementary Figure 1**). Age-standardized YLDs reached 486.9 (95% UI 337.6 to 664.1) per 100,000, a 35.4% increase (95% UI 29.1 – 41.3), and DALYs summed to 1023.6 (95% UI 870.9 to 1203.7) per 100,000, representing a minimal 3.1% (95% UI -8.2 to 1.7) decrease from 1990. Estimates of change in metrics for all ages, as shown in **Table 1**, were notably higher than those adjusted for age. Considering all disease causes, the GBD ranked T2DM as Brazil’s 3rd leading cause of disease burden in 2021, up from the 12^th^ leading cause in 1990 (data not shown).

**Supplementary Figure 2.**
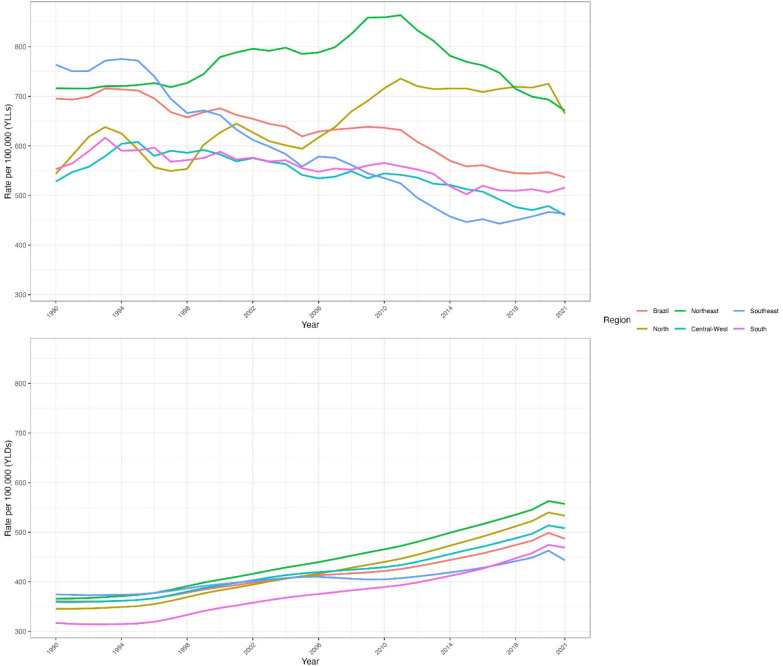
Years of life lost (YLL) and Years Lived with Disability (YLD) due to T2DM in Brazil and its macro-regions.

The Northeast region displayed the highest age-standardized mortality rate (32.2, 95% UI 29.3 to 34.3) per 100,000 habitants, 25% greater than that for Brazil overall, while the Central-West (22.2; 95% UI 19.9 to 23.8) and Southeast (22.1; 95% UI 19.7 to 23.6) presented the lowest mortality rates. The North region had a 26.1% increase, while deaths in Brazil overall decreased, especially in the Southeast region (-35%; 95% UI -38.9% to -31.2). As seen in **Table 1** and in **Supplementary Figure 2**, the same discordant trends occurred for YLLs. As shown by age-standardized YLDs, morbidity has consistently increased in all regions since 1990, with a tendency of slight decrease after 2020 observed in **Supplementary Figure 2**. The North region with a 53.4% (95% UI 46.3 to 60.7) increase, 50% greater than for Brazil overall. The North region also presented the most pronounced increase in DALYs, 34.8% (95% UI 27.2 to 41.4), compared to virtually no change for Brazil overall, and a 20.3% (95% UI -25.6 to -15.0) reduction in the Southeast region. In 2021, the Northeast region had the highest YLD (557.1; 95% UI 384.7 to 757.1) and DALY (1228.0; 95% UI 1051.5 to 1431.9) rates **(Table 1)**.

**Figure 1.**
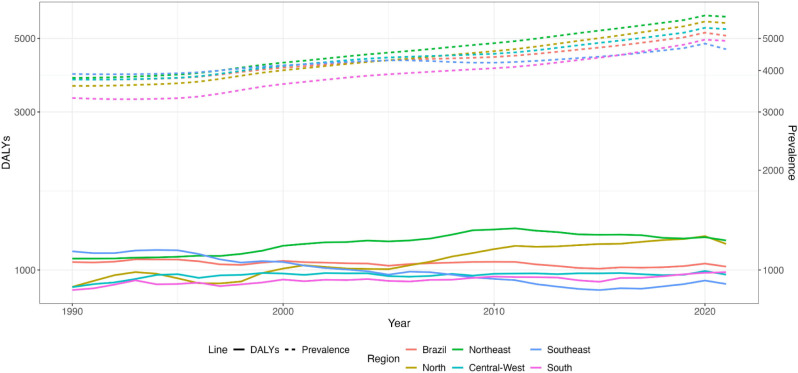
Prevalence and Disability-Adjusted Life Years (DALYs) lost to T2DM in Brazil and its macro-regions.

**Figure 1** graphically displays the change in age-standardized T2DM prevalence and DALYs in Brazil and its macro-regions between 1990 and 2021. The consistent increase in prevalence contrasts with the relatively stable course of DALYs over the study period. Slightly higher prevalences of DALYs are present in the North and Northeast regions, and lower ones are in the South and Southeast regions.

Dashed lines represent age-standardized prevalence rates, and the solid lines represent age-standardized DALYs rates per 100,000 habitants from 1990 to 2021.

**Figure 2.**
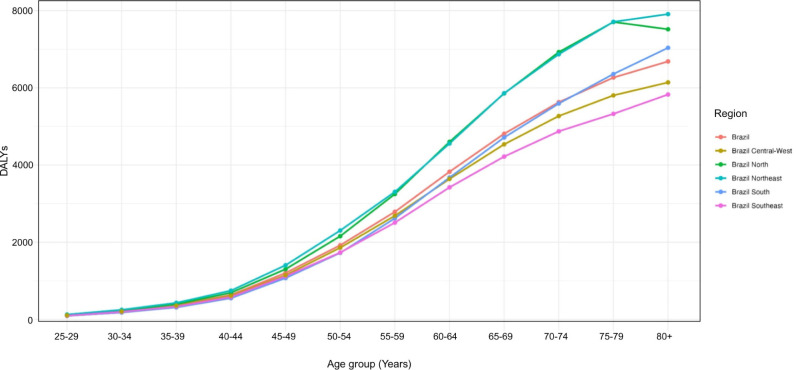
Disability-Adjusted Life Years (DALYs) of T2DM in Brazil and macro-regions in 2021, by age and region.

**Figure 2** shows Brazil’s DALY rates per 100,000 habitants and macro-regions, stratified by age group. Overall, DALY rates increased with age for all regions but with notable regional disparities. The North and Northeast consistently showed somewhat higher DALYs for all age groups, whereas the Central-West and Southeast regions had lower rates in almost all age groups.

The Y axis represents age-standardized DALYs, in rates per 100.000 habitants.

**Figure 3.**
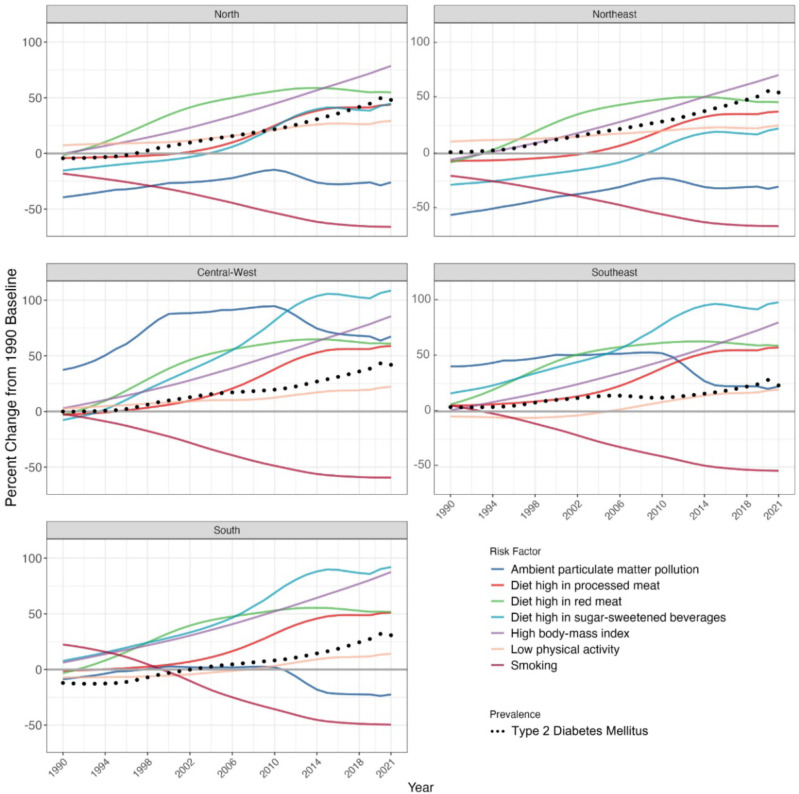
Change in age-standardized prevalence and risk factors’ exposure for T2DM in macro-regions.

**Figure 3** illustrates the change in age-standardized measures (1990–2021) for the prevalence of T2DM and its associated risk factors, with 1990 as the reference year for Brazil, disaggregated by region. A consistent increase in T2DM prevalence was observed across all regions, accompanied by varied but usually parallel changes in most diabetes risk factors. Consumption of sugar-sweetened beverages had the most pronounced increases in the Central-West, Southeast and South regions, followed by high BMI with a sharp and uniform increase nationwide. Increases in both these, high red meat and processed meat consumption were usually equal to or greater than those of prevalent diabetes. On the other hand, the exposure to smoking has decreased in all regions, and the more recently recognized risk factor, air pollution, increased in Central-West and Southeast, and reduced in other regions. Finally, low physical activity experienced smaller increases in all regions.

Data are presented as age-standardized percent change, from 1990 to 2021. The baseline for each risk factor is its SEV in 1990 for Brazil overall.

## DISCUSSION

In this study, we showed a large fatal and non-fatal burden of T2DM in Brazil in 2021. Between 1990 and 2021, the national age-standardized prevalence of T2DM increased by 37% and its incidence by 32%. Despite this surge in cases, age-standardized deaths decreased by 18% and YLLs by almost 23%, reflecting improved outcomes and advances in diabetes care; notably, this mortality reduction was more pronounced in females than in males. Conversely, age-standardized YLDs increased substantially by 35%, leading to only a modest 3% reduction in DALYs, suggesting that gains in preventing premature deaths are increasingly offset by a growing burden of living with the disease. Furthermore, while age-standardized rates showed declines in mortality, crude diabetes mortality, YLLs, and DALYs actually increased across Brazil over these three decades, primarily driven by population aging. Regionally, the Northeast presented slightly greater age-standardized frequency and burden in 2021, and the North experienced considerably larger increases in these metrics since 1990. Exposure to most diabetes risk factors, when age-standardized, also showed marked increases in all regions over the period, with sugar-sweetened beverage consumption, high BMI and red and processed meat consumption outstripping the increases in diabetes prevalence in most regions.

Increases in both crude and age-standardized prevalence and incidence related to T2DM in Brazil between 1990 and 2021 are by global trends, as age-standardized diabetes prevalence increased by more than 100% in almost half of the 204 countries and territories included in the GBD, and more than 90% of these diabetes cases were T2DM ^(20)^. This pattern has been observed in most low- and middle-income countries (LMICs) in a more rapid way compared to high-income countries, gradually shifting from environments leading infectious, childhood, and maternal diseases to be the principal public health concerns to ones with a higher burden from non-communicable diseases ^(22)^. For example, International Diabetes Federation (IDF) estimated that 94% of the increase in the number of people with diabetes by 2045 will occur in low and middle-income countries ^(23)^.

In contrast, significant reductions in age-standardized mortality rates for T2DM and YLL were observed in Brazil, with more pronounced declines in the Southeast and Central-West. Alongside regional disparities, clear sex-specific patterns were observed, with females experiencing a more favorable trend in T2DM mortality with greater reductions over time than males. These findings indicate that the improvements have not occurred uniformly across geographic regions or between sexes, highlighting differences in behavioral susceptibility to health risks ^(24)^ and heterogeneous impact of health system advances across the country. These trends likely reflect not only global advances in evidence-based care of diabetes and the management of complications, notably the cardiovascular ones, but also, specifically in Brazil, the expansion of primary care and improvement across all levels of the health system following the creation and consolidation of the Unified Health System ^(25,26)^. These actions have blunted the rise in morbimortality accompanying the rapid increase in diabetes cases. Since 2007, the right to receive free anti-diabetic medications and related treatment supplies (e.g., syringes) has been guaranteed to people living with diabetes in Brazil, with a major increase in access to free diabetes medications starting in 2011 ^(27)^. In 2019, the Brazilian Congress enacted the National Policy for Diabetes Prevention and Comprehensive Assistance to Diabetic Persons was established, aiming to increase population awareness about diabetes detection and treatment.

Besides some advances in the Brazilian health system and treatment policies, increases in both crude and age-standardized fatal and non-fatal burden of T2DM were observed in the Northeast and North, in contrast to the Southeast region, exacerbating regional Brazilian health disparities. The North and Northeast regions suffer from poorer health outcomes due to historical, socioeconomic, and structural factors, with key challenges such as elevated poverty levels, restricted access to quality healthcare services, and lower levels of educational attainment ^(28)^. These challenges within specific settings should be addressed to reduce regional health inequalities and improve public health outcomes ^(29-31)^.

Additionally, deferred care-seeking during the COVID-19 pandemic may have affected T2DM morbidity and mortality across Brazilian regions, as poorer health-care infrastructures were less equipped to manage infections and the increased demand for health services. Although GBD 2021 estimates were adjusted for the impact of COVID-19, it is not possible to fully evaluate the pandemic’s specific contribution to T2DM-related burden. In our study, annual trends in prevalence, DALYs, and YLLs after 2019 remained consistent with those observed in previous years across all regions. However, age-standardized YLDs showed a reduction trend after 2019-2020 in all regions, which may be reflecting an under-identification of T2DM complications due to the pandemics’ impact on medical appointments ^(32)^. These trends should be monitored in future GBD iterations, and targeted efforts within health-care services are needed to recover delayed services resulting from the pandemic period.

Finally, we showed that diabetes risk factor control, except for tobacco, has, in large part, worsened over the last three decades in Brazil, contributing to its increasing incidence and prevalence. Over the past three decades, Brazil’s socioeconomic and urban development has been accompanied by significant societal changes. Notably, there has been an increase in the consumption of ultra-processed foods and a rise in dense urbanization that offers limited recreational space ^(33-35)^. This urban growth is coupled with challenges such as inadequate sidewalks, poor lighting, and crime, hindering regular physical activity, exacerbating many risk factors, and impeding substantial improvements in others, such as promoting physical activity ^(35)^. These changes appear to be the motor of the current diabetes epidemic. However, other less well-described factors related to environmental pollution resulting from societal change may also play a significant role ^(36)^, as well as the increased exposure to endocrine-disrupting chemicals in ultra-processed products or their packaging ^(37,38)^.

The World Health Organization (WHO) provided a list of interventions, including population-based ones, considered to be the most cost-effective means of preventing chronic diseases such as diabetes, including 1) increasing prices and taxes on tobacco and alcohol products, 2) promoting awareness through mass media on the importance of healthy diets and physical activity, 3) Reducing salt consumption and salt content in food and replacing trans fats by polyunsaturated fats, and/or restrict the marketing of food and beverage of these products, especially for children ^(39)^. Despite the attractiveness of these multisectoral and population-based approaches, implementation in Brazil, like in most countries, has been partial. Recent analyses suggest that, despite improvements in clinical care, the goals set by the United Nations for the year 2030 concerning chronic diseases, including diabetes, will not be reached ^(40)^. The inability of the Brazilian government and society, either unaware of the impact of these underlying actors or unable to direct social development in a healthy direction, has produced this major public health failure. The Strategic Action Plans to Tackle Non-Communicable Diseases (NCDs) in Brazil (2011-2022 and 2021-2030) were created to cope with and restrain NCD risk factors in the next 10 years ^(41,42)^. However, evidence regarding the effectiveness of its policies remains limited.

This study acknowledges several limitations that are inherent to dealing with modelled and secondary data. First, while Brazilian mortality data is generally reliable, deficiencies in the Brazilian national mortality information system in earlier years of the GBD series add uncertainty to the trends in the mortality and burden metrics we report, especially for the North and Northeast. Second, we were unable to assess trends in specific causes of death among individuals with T2DM because the GBD relies on the underlying cause recorded on death certificates. This means that when T2DM is listed as the cause of death, it is not possible to determine whether it resulted, for example, from cardiovascular disease or other conditions. Third, estimating diabetes prevalence also poses challenges due to its reliance on laboratory data as required by the GBD framework. Being based on only 17 Brazilian studies into models initially oriented by global data, our estimates of prevalence, incidence, and YLDs are especially subject to error. Finally, the assumption that all deaths under the age of 15 are related to T1DM can produce a misclassification bias, and under- or overestimations in T1DM metrics can influence our results, as the non-fatal burden of T2DM is obtained by removing that of T1DM from the total diabetes mellitus.

Nonetheless, a significant strength of our study is the use of up-to-date, internationally comparable disease metrics derived from rigorous and sophisticated GBD analyses to assess the burden and trends of T2DM. By considering the best available epidemiological evidence and controlling for different sources of bias, the wide 95% uncertainty intervals presented in the tables indicate the large degree of uncertainty present. However, it provides a straightforward synthesis of the current T2DM scenario in Brazil, instrumental in guiding the planning, implementation, and evaluation of public policies to mitigate the burden of this condition.

In conclusion, the burden of T2DM in Brazil has significantly increased from 1990 to 2021, most notably in the North and Northeast regions. Addressing disparities requires targeted interventions that improve healthcare access, enhance care integration, and expand educational programs. Unless prevention strategies that address key risk factors such as excessive weight, sedentary behavior and an unhealthy diet are implemented, the burden of T2DM will likely continue to grow at an accelerated pace.

## Figures and Tables

The lines represent age-standardized YLL and YLDs rates per 100,000 habitants from 1990 to 2021.

## Data Availability

the data supporting this study’s findings are publicly available online for download from the GBD Results Tool (http://ghdx.healthdata.org/gbd-results-tool).
